# Comparative Effectiveness of Clopidogrel Versus Aspirin for Primary Prevention in High-Risk Patients with Type 2 Diabetes: A Nationwide Propensity Score–Matched Cohort Study

**DOI:** 10.3390/medicina61101730

**Published:** 2025-09-23

**Authors:** Soo Hyun Kang, Joonpyo Lee, Jung Ho Kim, Youngwoo Jang

**Affiliations:** 1 Gachon Medical Research Institute, Gachon Biomedical Convergence Institute, Gachon University Gil Medical Center, Incheon 21565, Republic of Korea; ksh.copo@gmail.com; 2 Department of Research and Analysis, National Health Insurance Service Ilsan Hospital, Goyang 10444, Republic of Korea; 3 Division of Cardiology, Gachon University Gil Medical Center, College of Medicine, Gachon University, Namdong-daero 774, Namdong-gu, Incheon 21565, Republic of Korea; joonpyu@gilhospital.com; 4 Department of Translational-Clinical Medicine, Gachon Advanced Institute for Health Sciences and Technology (GAIHST), Gachon University, Incheon 21999, Republic of Korea; 5 Division of Gastroenterology, Department of Internal Medicine, Gachon University Gil Medical Center, College of Medicine, Gachon University, Incheon 21565, Republic of Korea

**Keywords:** aspirin, clopidogrel, diabetes mellitus, bleeding, primary prevention

## Abstract

*Background and Objectives*: The benefits of aspirin for primary prevention of atherosclerotic cardiovascular disease (ASCVD) among high-risk patients with diabetes are controversial owing to bleeding risk. Current guidelines recommend the use of aspirin in high-risk patients with diabetes; however, the supporting evidence is inconsistent, primarily due to an increased risk of gastrointestinal (GI) bleeding. Given these concerns, it is important to explore alternative antiplatelet strategies. Clopidogrel, a widely used P2Y12 inhibitor, has been suggested to cause fewer GI bleeding events than aspirin. Accordingly, we aimed to compare the efficacy and bleeding risk of clopidogrel versus aspirin in high- and very high-risk populations with diabetes without prior ASCVD using the Korean National Health Insurance Service data. *Materials and Methods*: Propensity score-matching was performed to reduce baseline imbalances. The primary endpoint was net adverse clinical events (NACEs), defined as a composite of all-cause death, myocardial infarction (MI), stroke, intracranial hemorrhage (ICH), and gastrointestinal GI bleeding. Secondary endpoints included efficacy (composite of all-cause death, MI, and stroke) and bleeding outcomes (GI bleeding and ICH). *Results*: Among 10,453 patients (9550 on aspirin and 903 on clopidogrel), 902 matched pairs were analyzed. Clopidogrel showed no significant difference compared with aspirin in NACE incidence (hazard ratio [HR]: 0.97; 95% confidence interval [CI]: 0.79–1.19), efficacy endpoints (HR: 1.02; 95% CI: 0.82–1.26), or individual outcomes (MI, stroke, all-cause death). Clopidogrel demonstrated a trend towards lower GI bleeding (HR: 0.48; 95% CI: 0.23–1.01), although not significant. In subgroup analysis, male patients on clopidogrel had significantly lower NACE risk than those on aspirin (HR: 0.73; 95% CI: 0.54–0.99). *Conclusions*: These findings suggest that clopidogrel may be considered a preferable alternative to aspirin for primary prevention in high-risk male patients with diabetes, particularly those with an elevated risk for gastrointestinal bleeding, guiding personalized antiplatelet therapy choices in clinical practice.

## 1. Introduction

Atherosclerotic cardiovascular disease (ASCVD) is a leading cause of morbidity and mortality worldwide, particularly among patients with diabetes who have an elevated cardiovascular risk even in the absence of prior events [1,2,3,4,5]. Numerous studies have identified the risk factors for ASCVD, among which diabetes is a major contributor to its development [6]. In addition to traditional risk factors, gender can act as a critical variable influencing both ASCVD risk and treatment response; for example, the net clinical benefit of antiplatelet therapy such as aspirin may vary between men and women [7,8]. To reduce ASCVD burden in such high-risk groups, a multi-dimensional approach is essential [9]. The successful implementation of these complex treatment regimens necessitates a multi-disciplinary, team-based model of care that incorporates physicians, pharmacists, physical therapists, dietitians and advanced practice nurses [10,11,12]. Moreover, ensuring the successful implementation of such strategies requires adequate financial investment and robust healthcare resources to guarantee both accessibility and sustainability [13,14]. Within this comprehensive framework, pharmacological therapy and interventional strategies occupy a central role, with preventive agents and interventional strategies have been consistently demonstrated to reduce mortality [15,16,17,18,19]. However, while the role of aspirin in the secondary prevention of ASCVD is well established, its role in primary prevention remains uncertain [20,21].

Several studies have attempted to clarify the role of aspirin in primary prevention, including three major studies published in 2018: Aspirin in Reducing Events in the Elderly (ASPREE), Aspirin to Reduce Risk of Initial Vascular Events (ARRIVE), and A Study of Cardiovascular Events in Diabetes (ASCEND) [22,23,24]. These trials have investigated the net benefit of aspirin in primary prevention and discovered marginal reductions in ischemic events that are offset by an increased risk of bleeding, particularly gastrointestinal bleeding. Based on these findings, in primary prevention settings, current guidelines only recommend aspirin for patients with high-risk ASCVD aged 40–70 years, due to bleeding risk considerations, especially among those with diabetes [25].

Clopidogrel, a P2Y12 inhibitor of the thienopyridine class, is widely used in the secondary prevention of ASCVD. Notably, clopidogrel has demonstrated superior profiles compared to aspirin in secondary prevention trials such as Clopidogrel versus Aspirin in Patients at Risk of Ischemic Events (CAPRIE) and Harmonizing Optimal Strategy for Treatment of Coronary Artery Stenosis–Extended Antiplatelet Monotherapy (HOST-EXAM), including lower gastrointestinal bleeding rates [26,27]. However, its role in primary prevention remains unclear, particularly among real-world populations with diabetes who are at an increased risk of ASCVD.

To address this knowledge gap, we investigated the comparative effectiveness and safety of clopidogrel versus aspirin in a large, real-world cohort of high- and very high-risk patients with diabetes without prior ASCVD using data from the Korean National Health Insurance System (NHIS) National Sample Cohort (NHIS-NSC). We aimed to assess the effectiveness and safety of clopidogrel compared with aspirin for primary prevention in this high-risk but understudied population.

## 2. Materials and Methods

### 2.1. Data Source and Study Population

We used data from the NHIS–NSC, managed by the Korean NHIS, which encompasses all residents of Korea [28,29,30,31]. The NHIS–NSC consists of approximately one million individuals randomly sampled from the total population enrolled in the health insurance and medical benefit programs in Korea, representing approximately 2.2% of the Korean population between 2002 and 2019.

The study population included patients with diabetes who were prescribed single antiplatelet therapy with either aspirin or clopidogrel for at least 90 consecutive days between 2010 and 2019. diabetes was defined based on either the prescription of antidiabetic medications or fasting blood glucose levels ≥ 126 mg/dL. To focus exclusively on primary prevention, we excluded individuals with a history of coronary artery disease, percutaneous coronary intervention (PCI) procedures, stroke, or peripheral artery disease. Coronary artery disease was defined as hospitalization with a diagnosis coded I20–I22 under the International Classification of Diseases, 10th revision (ICD-10). Stroke was defined as hospitalization with a diagnosis of ICD-10 codes I63–I64 accompanied by brain imaging with computed tomography (CT) or magnetic resonance imaging (MRI) performed within 90 days. Peripheral artery disease was identified by the ICD-10 code I73.9. Patients with incomplete demographic data were also excluded from the study. The dataset was named Clopidogrel versus AsPirin as a STrategy fOr the PreventioN of cardiovascular diseasEs in patients with Diabetes Mellitus (CAPSTONE-DM).

The CAPSTONE-DM study was granted an exemption from review by the Institutional Review Board of Gachon University Gil Medical Center, Incheon, Korea (IRB exemption number: GFIRB2023-459) as it involved a retrospective analysis of anonymized administrative data. Accordingly, the requirement for informed consent was waived. Data were accessed from 10 October 2024, to 15 April 2025. All data were fully anonymized and contained no personal health information, precluding individual identification.

This study was reported in accordance with the STROBE (Strengthening the Reporting of Observational Studies in Epidemiology) guidelines.

### 2.2. Definition and Risk Assessment of DM

In the CAPSTONE-DM cohort, only individuals categorized as high-risk or very high-risk patients with diabetes were included [32,33]. Patients classified as high-risk were defined as having diabetes for ≥10 years, accompanied by at least one additional factor, including age ≥ 50 years, hypertension, dyslipidemia (defined as an ICD-10 code diagnosis of E78, ongoing statin therapy, or low-density lipoprotein [LDL] cholesterol levels ≥ 130 mg/dL), current smoking status, or body mass index (BMI) ≥ 25 kg/m^2^. Patients in the very high-risk group were defined as having either diabetes-related target organ damage or at least three of the aforementioned conditions [32,33]. Target organ damage encompassed diabetic retinopathy (ICD-10 codes H36.0, E10.3-E14.3) [34], diabetic neuropathy (diagnosed via ICD-10 codes E10.4-E14.4, G59.0, G63.2, G99.0 or co-prescription of glucose-lowering medications [anatomical therapeutic chemical codes A10A/A10B] and diabetic retinopathy medications [anatomical therapeutic chemical codes A16AX01, D11AX02, N06AA10, N06AA09, N03AX12, N03AX16, N06AX21]) [35], and diabetic nephropathy (ICD-10 codes E10.2-E14.2 or N08.3) [36]. Baseline insulin use was defined as insulin treatment exceeding 3 months within the year preceding aspirin or clopidogrel initiation.

### 2.3. Aspirin and Clopidogrel Therapy

We identified patients who consistently received either aspirin or clopidogrel as single antiplatelet therapy for a minimum of 90 days. Drug exposure was tracked using the active substance codes aspirin (1107 and 1110) and clopidogrel (4925, 4988, 5015, 1369, and 5179).

### 2.4. Definition of Other Comorbidities and Laboratory Data

Hypertension was defined based on an established diagnosis (ICD-10 code I10), prescription of antihypertensive medication, or measured blood pressure ≥ 140/90 mmHg during health checkups. Laboratory and clinical data, including blood pressure, fasting glucose levels, BMI, LDL cholesterol, high-density lipoprotein (HDL) cholesterol, triglyceride, creatinine, and hemoglobin levels, were extracted from the National Health Examination records collected within 2 years prior to the initiation of antiplatelet therapy. LDL cholesterol levels were estimated using the Friedewald equation [37,38,39]. Missing data from the National Health Examination were imputed using mean values within each diabetic risk stratification group. Statin therapy, including statin–ezetimibe combination therapy, was defined as statin use for >3 months preceding the initiation of antiplatelet treatment.

### 2.5. Clinical Follow-Up and Definition of Endpoints

The primary endpoint was net adverse clinical events (NACEs), a composite outcome that encompassed both efficacy and bleeding complications. Secondary endpoints included the efficacy endpoints (combined all-cause death, myocardial infarction [MI], and stroke) and bleeding endpoints (gastrointestinal [GI] bleeding and intracranial hemorrhage [ICH]). The individual secondary endpoints included all-cause death, MI, stroke, GI bleeding, and ICH. Event identification utilized the ICD-10 and Medical Care Procedure codes MI (I21), stroke (hospitalization for I63-I64 and CT/MRI within 90 days), ICH (hospitalization or outpatient treatment for I60-I62 with associated transfusion), and GI bleeding. GI bleeding was specifically defined as a history of endoscopic hemostasis, vascular embolization procedures, or transfusions coupled with the specified ICD-10 and endoscopic procedure codes (E7611, EZ937, E7660, E7670, and E7680) within 30 days of transfusion. Relevant ICD-10 codes for GI bleeding included K26.0, K26.2, K26.4, K26.6, K27.0, K27.2, K27.4, K27.6, K28.0, K28.2, K28.4, K28.6, K29.0, K62.0, K92.1, and K92.2. Follow-up continued until the initial NACE or the end of the study period (31 December 2019). Patients who experienced NACEs within 90 days of starting medication or those with a therapy duration < 90 days were excluded to minimize reverse causation.

### 2.6. Statistical Analysis

Propensity score matching (PSM) was conducted at a 1:1 ratio (caliper width, 0.2) to balance the baseline characteristics of the aspirin and clopidogrel groups. Covariates in the matching included gender, age, hypertension, diabetic retinopathy, neuropathy, nephropathy, atrial fibrillation, heart failure, statin therapy, fasting glucose level, BMI, diabetes duration, and antiplatelet treatment.

Baseline characteristics were compared using chi-square tests, *t*-tests, and analysis of variance based on antiplatelet medication status. Hazard ratios (HRs) and 95% confidence intervals (CIs) were calculated using Cox proportional hazard regression models after adjusting for covariates. Kaplan–Meier survival curves were used to assess the incidence of NACEs across the medication groups. Statistical significance was set at *p* < 0.05. Statistical analyses were performed using SAS Enterprise version 8.3 (SAS Institute, Cary, NC, USA) and R version 4.3.0 (R studio, PBC).

### 2.7. Data and Resource Availability

The datasets analyzed in the current study can be requested from the NHIS upon reasonable justification (https://nhiss.nhis.or.kr/en/z/a/001/lpza001m01en.do, accessed from 10 October 2024, to 15 April 2025).

## 3. Results

### 3.1. Baseline Characteristics

**Figure 1:**
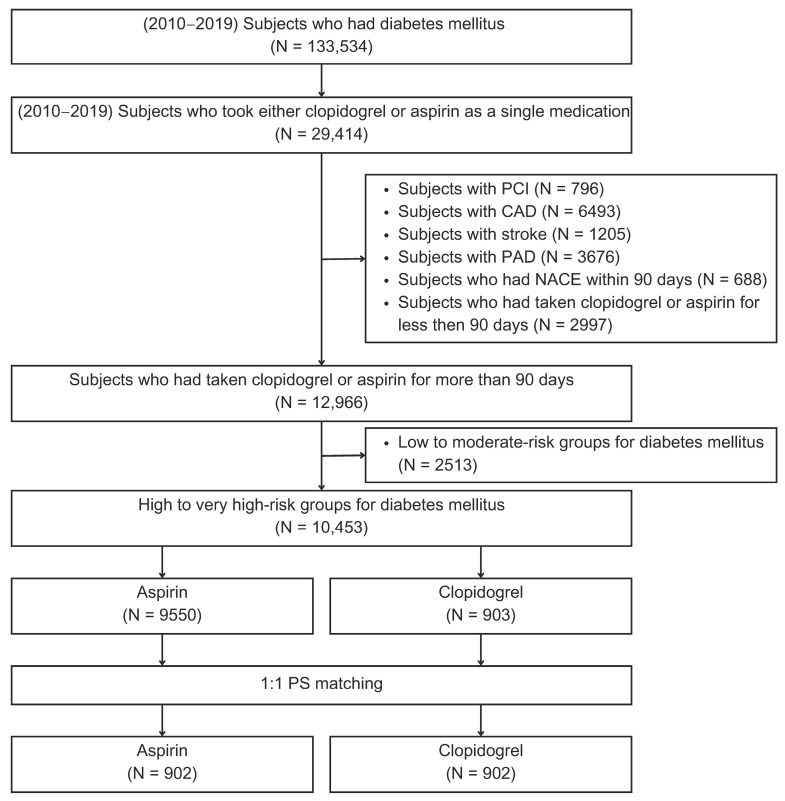
Study design and population selection for the CAPSTONE-DM study. From a Korean National Health Insurance Service–National Sample Cohort (2010–2019), 133,534 diabetic patients were screened. After applying multiple exclusion criteria and identifying high-/very high-risk diabetic patients without ASCVD, 903 patients on clopidogrel and 9550 on aspirin were selected. Propensity score matching (1:1) yielded 902 patients in each group.

From the 1 million individuals in the NHIS-NSC, we identified 133,534 individuals diagnosed with diabetes between 2010 and 2019 for this study (Figure 1). Of these, 29,414 received either clopidogrel or aspirin as a single-antiplatelet therapy. After selecting those who did not meet the exclusion criteria, we identified 12,966 participants who had taken clopidogrel or aspirin for primary prevention. We further selected 10,453 patients who were eligible for the high- or very high-risk categories for diabetes, of whom 9550 and 903 were on aspirin and clopidogrel, respectively. After 1:1 PSM, 902 patients remained in each group, allowing for a balanced comparison.

The baseline characteristics of the patients before and after PSM are presented in Table 1. The mean age of the patients was approximately 66 years (male gender, 51%), with no significant differences in age or gender distribution between the groups after PSM. Clinical characteristics, such as the prevalence of hypertension, BMI, atrial fibrillation, and heart failure, were well balanced between the groups after PSM. However, significant differences remained even after PSM. The duration of diabetes was approximately 5 months longer in the clopidogrel group than that in the aspirin group (*p* < 0.0001). Furthermore, the prevalence of chronic kidney disease in the clopidogrel group was >2-fold higher than that in the aspirin group (*p* = 0.0026).

### 3.2. Outcome Analysis

**Figure 2:**
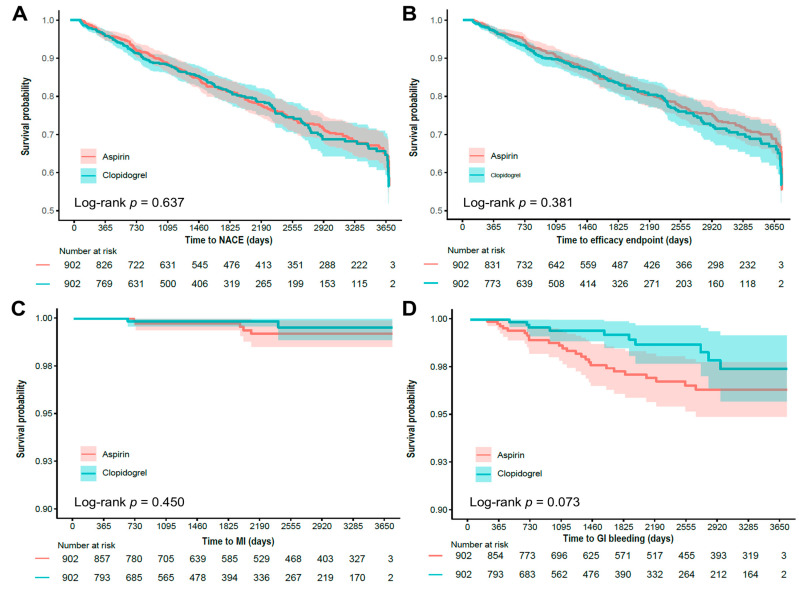
Kaplan–Meier survival curves for clinical outcomes in propensity score–matched patients. (**A**) Time to net adverse clinical event (NACE), defined as a composite of GI bleeding, ICH, all-cause death, MI, and stroke; (**B**) Time to efficacy endpoint (MI or stroke); (**C**) Time to MI; (**D**) Time to GI bleeding. *p* values from log-rank tests are shown.

Table 2 presents a comparison of the primary and secondary endpoints between the aspirin and clopidogrel groups, with aspirin used as the reference group for all HR calculations. For the primary endpoint NACE, no significant difference was observed between the aspirin and clopidogrel groups after PSM (HR: 0.97; 95% CI: 0.79–1.19). No difference was observed in the efficacy endpoint between groups (HR: 1.02; 95% CI: 0.82–1.26) and secondary endpoints, including MI (HR: 0.52; 95% CI: 0.10–2.83), stroke (HR: 1.22; 95% CI: 0.60–2.48), and all-cause death (HR: 1.07; 95% CI: 0.86–1.33) (Table 2 and Figure 2A–C). Regarding bleeding outcomes, a trend towards a lower incidence of GI bleeding was observed in the clopidogrel group (HR: 0.48; 95% CI: 0.23–1.01, Table 2 and Figure 2D). The incidence of ICH was similar between the two groups (HR: 1.36; 95% CI: 0.64–2.88).

### 3.3. Subgroup Analysis

**Figure 3:**
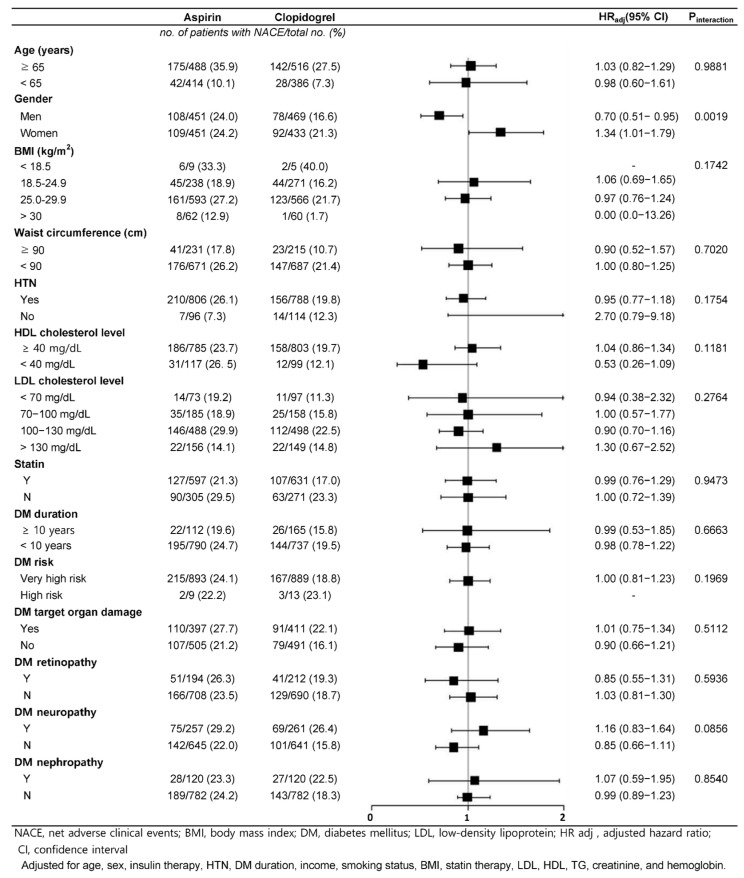
Subgroup analysis of net adverse clinical events (NACEs) comparing clopidogrel versus aspirin. Forest plot shows adjusted hazard ratios (HRs) with 95% confidence intervals (CIs) for prespecified subgroups. NACE, net adverse clinical event; BMI, body mass index; DM, diabetes mellitus; LDL, low-density lipoprotein; HR adj, adjusted hazard ratio; CI, confidence interval.

Subgroup analyses based on various baseline characteristics, including age, gender, BMI, and diabetes duration, did not reveal any significant interaction effects for the primary endpoint, suggesting that the comparative efficacy and safety of clopidogrel versus aspirin were consistent across different patient subgroups (Figure 3). Notably, a significant interaction was observed for gender, with men showing a lower HR for NACEs when treated with clopidogrel compared with aspirin (HR: 0.73; 95% CI: 0.54–0.99, p-interaction = 0.0015).

## 4. Discussion

In this study, we analyzed the net clinical benefits of aspirin and clopidogrel in high- and very high-risk patients with diabetes without prior ASCVD. Our results revealed that clopidogrel did not significantly reduce NACEs compared with aspirin. No significant differences were observed in efficacy outcomes, including MI, stroke, and all-cause death, between the two groups. However, clopidogrel demonstrated a non-significant trend towards a lower incidence of GI bleeding, which may indicate a potential safety advantage in terms of GI complications. While our primary analysis did not demonstrate the superiority of clopidogrel over aspirin for NACEs, this neutral finding itself is clinically significant. It suggests that clopidogrel provides comparable efficacy for the prevention of major adverse events, positioning it as a viable alternative for patients in whom aspirin may be suboptimal, such as those at high risk for gastrointestinal complications. The results of subgroup analysis suggested that men might particularly benefit from clopidogrel treatment over aspirin for primary prevention.

Current guidelines for the primary prevention of cardiovascular diseases increasingly limit aspirin use. The 2019 American College of Cardiology/American Heart Association Guideline on the Primary Prevention of Cardiovascular Disease recommends against the use of aspirin in adults aged ≥ 70 years and suggests that aspirin may be considered in high-risk adults aged 40 to 70 years (Class IIb) [11]. The 2023 American Diabetes Association Guidelines on Standards of Medical Care in Diabetes also recommend the use of aspirin for patients with diabetes and a high risk of ASCVD (Class IIb) but not for those with a low risk (Class III) [40]. In addition, clopidogrel is recommended for primary prevention only in individuals who are unable to tolerate aspirin because of allergies. Given the increased bleeding risks associated with aspirin, particularly GI bleeding, considerable interest has grown in identifying alternative antiplatelet agents with comparable efficacy but improved safety profiles [41]. Clopidogrel has emerged as a potential alternative, offering similar anti-ischemic benefits while potentially reducing GI complications.

Although no large randomized trials have directly evaluated the effectiveness of clopidogrel for primary prevention, insights can be obtained from comparative studies conducted in secondary prevention settings. The HOST-EXAM trial directly compared aspirin and clopidogrel as single-antiplatelet therapies in patients who completed 6–18 months of dual antiplatelet therapy without adverse events after PCI [27]. In this randomized trial, clopidogrel monotherapy significantly reduced the risk of the primary composite outcome—comprising all-cause death, nonfatal MI, stroke, readmission due to acute coronary syndrome, and Bleeding Academic Research Consortium (BARC) type 3 or greater bleeding—compared to aspirin monotherapy (5.7% vs. 7.7%; HR: 0.73; 95% CI: 0.59–0.90; *p* = 0.003). Clopidogrel also resulted in a lower risk of bleeding (BARC type ≥ 2: 2.3% vs. 3.3%; HR: 0.70; 95% CI: 0.51–0.98; *p* = 0.036). A meta-analysis comparing clopidogrel and aspirin in patients undergoing PCI also showed that clopidogrel was associated with a lower risk of ischemic events (risk ratio [RR]: 0.77; 95% CI: 0.65–0.91), any stroke (RR: 0.51; 95% CI: 0.35–0.76), ischemic stroke (RR: 0.55; 95% CI: 0.32–0.94), and hemorrhagic stroke (RR: 0.24; 95% CI: 0.09–0.68) compared to aspirin [42]. However, for major bleeding, clopidogrel showed a trend towards a lower risk compared to aspirin, albeit not significant (RR: 0.74; 95% CI: 0.43–1.29). In patients with stable coronary artery disease, a previous meta-analysis found no significant differences between clopidogrel and aspirin for composite endpoints (cardiovascular death, MI, and stroke) (odds ratio [OR]: 0.99, 95% CI: 0.47–2.10) or bleeding (BARC type 3 or above) (OR: 1.28, 95% CI: 0.78–2.12) [43]. A meta-analysis comparing clopidogrel and aspirin in patients with ischemic stroke also showed that clopidogrel was associated with a lower risk of major adverse cardiovascular and cerebrovascular events (RR: 0.72; 95% CI: 0.53–0.97) and a lower risk of bleeding (RR: 0.57; 95% CI: 0.45–0.74) [44]. Although results vary depending on patient populations and endpoints, these findings collectively suggest that clopidogrel may offer a favorable safety profile relative to aspirin, which could be relevant when considering primary prevention strategies, particularly in high-risk populations such as patients with diabetes.

Aspirin has the propensity to cause increased GI bleeding through multiple mechanisms. As an irreversible cyclooxygenase-1 inhibitor, aspirin reduces thromboxane A2-mediated platelet aggregation and impairs gastric mucosal defense by suppressing protective prostaglandin synthesis, thereby increasing the susceptibility to mucosal injury and bleeding. Several large-scale meta-analyses have demonstrated that while aspirin may modestly reduce the incidence of first cardiovascular events in primary prevention, this benefit is often offset, or even outweighed by a significantly increased risk of major bleeding, resulting in no clear net clinical advantage [21,45]. This bleeding-prone pharmacologic profile has prompted the exploration of alternative or modified strategies. In high-risk patients with diabetes without prior ASCVD, our recent analysis demonstrated that compared with aspirin, the use of sarpogrelate was associated with reduced GI bleeding and improved net clinical outcomes [41]. Similarly, in the recent effect of short Duration of DAPT followed by Dose reduction after Implantation of DCS in ACS patients (4D-ACS) trial involving patients with acute coronary syndrome, we demonstrated that early discontinuation of aspirin, followed by P2Y12 inhibitor monotherapy, led to a marked reduction in bleeding events, most of which were gastrointestinal [16]. Other randomized trials have shown increased GI bleeding with aspirin use among elderly or moderate-risk individuals, without a corresponding reduction in ischemic outcomes [46]. Collectively, these findings suggest that aspirin-associated GI bleeding may attenuate the net clinical benefit of aspirin in certain subgroups, highlighting the potential advantages of alternative antiplatelet strategies such as clopidogrel for primary prevention.

In the subgroup analysis, clopidogrel use in men was associated with a lower incidence of NACEs compared to aspirin (HR: 0.73; 95% CI: 0.54–0.99; p for interaction = 0.0015). This finding has been reported in other studies and may suggest a potential gender-specific treatment effect. Previous studies have also reported gender-related differences in response to clopidogrel. For example, among patients with acute coronary syndrome, women have a higher 1-year risk of atherothrombotic events than men, particularly female carriers of the CYP2C9*3 loss-of-function allele, indicating a possible gender-by-genotype interaction in clopidogrel responsiveness [47]. Similarly, a post hoc gender analysis of the HOST-EXAM trial showed no significant differences between clopidogrel and aspirin in women, whereas clopidogrel was associated with lower rates of both ischemic and bleeding events in men [48]. While the frequency of the CYP2C19 loss-of-function alleles may not differ by gender, women have been shown to exhibit higher on-treatment platelet reactivity in response to clopidogrel. In one study, women undergoing cardiac surgery demonstrated significantly greater ADP-induced platelet aggregation and a higher rate of high platelet reactivity when the platelet count exceeded 200,000/μL (45.5% vs. 11.9%) [49]. These findings suggest that gender-related differences in pharmacodynamics, which are potentially influenced by platelet count, hormonal factors, and drug metabolism, may contribute to the observed differential efficacy of clopidogrel between men and women. Beyond pharmacological interventions, it is also important to consider the translational perspective of lifestyle modifications on vascular health, particularly in women [50].

### 4.1. Perspective for Clinical Practice

The findings from this study support consideration of a more personalized antiplatelet strategy for primary prevention in high-risk patients with diabetes. The neutral primary outcome is clinically significant in that it establishes clopidogrel as a plausible alternative to aspirin. This finding encourages a nuanced approach where patient selection is guided by individual risk profiles. For instance, in patients with a high risk of GI bleeding, the trend toward a better safety profile with clopidogrel, though not reaching statistical significance, may warrant consideration. Most compellingly, the significant reduction in NACEs among men is a noteworthy finding that suggests a potential benefit in this subgroup. Thus, clinical decisions should be individualized, carefully balancing a patient’s ischemic and bleeding risks while treating gender as a potentially critical factor in the therapeutic equation. Ultimately, the selection of an antiplatelet agent should be viewed as one component of a comprehensive, multi-disciplinary care plan. The clinical benefit of a personalized antiplatelet strategy is maximized when integrated with the meticulous control of other risk factors, achieved through a collaborative, multi-disciplinary team effort [9,10,51].

### 4.2. Limitation

This study had several limitations. First, as a retrospective study, it was subject to inherent biases, including selection bias and unmeasured confounding factors. This is particularly relevant given the significant initial imbalance between the aspirin (N = 9550) and clopidogrel (N = 903) groups, which may reflect underlying selection biases that cannot be fully resolved by propensity score matching. Additionally, factors that may influence GI bleeding, such as the use of proton-pump inhibitors, were not excluded or controlled for in the analysis. Second, since the study focused on patients with diabetes with high or very high ASCVD risk, the findings may not be generalizable to the general populations with diabetes. Third, despite using PSM to balance patient characteristics, significant differences in the duration of diabetes and the prevalence of chronic kidney disease persisted between the groups after matching. These differences may still influence clinical outcomes and should be interpreted cautiously when generalizing our results. Finally, the study may have been underpowered to detect statistically significant differences in low-frequency outcomes such as GI bleeding. Future prospective studies are warranted to confirm these findings and evaluate the optimal antiplatelet strategy in high-risk patients with diabetes without established ASCVD.

## 5. Conclusions

In high- or very high-risk patients with diabetes without prior ASCVD, clopidogrel did not differ significantly from aspirin in terms of the incidence of NACEs. However, clopidogrel showed a trend towards a lower risk of GI bleeding, although not statistically significant. Notably, the subgroup analysis demonstrated a significant reduction in NACEs among male patients treated with clopidogrel compared with those treated with aspirin. These findings suggest that clopidogrel could be considered an alternative to aspirin for primary prevention, especially in patients with diabetes at increased risk for gastrointestinal complications, particularly in men. Individual risk assessments should guide clinical decision-making regarding antiplatelet therapy choices.

## Figures and Tables

**Table 1:** Baseline characteristics.

	Pre-Matched				Post-Matched			
	Total (N = 10,453)	Aspirin (N = 9550)	Clopidogrel (N = 903)	*p*	Total (N = 1804)	Aspirin (N = 902)	Clopidogrel (N = 902)	*p*
Demographic data								
Age (years)	63 ± 11	62 ± 11	66 ± 11	0.1587	66 ± 11	66 ± 11	66 ± 11	0.2095
Male, N (%)	5876 (56.2)	5406 (56.6)	470 (52.1)	0.0092	920 (51.0)	451 (50.0)	469 (52.0)	0.4233
Body mass index (kg/m^2^)	26 ± 3	26 ± 3	26 ± 3	0.4455	26 ± 3	26 ± 3	26 ± 3	0.5383
<18.5	54 (0.52)	49 (0.5)	5 (0.6)	0.7849	14 (0.8)	9 (1.0)	5 (0.6)	0.2676
18.5–24.9	3000 (28.7)	2729 (28.6)	271 (30.0)		509 (28.2)	238 (26.4)	271 (30.0)	
25.0–29.9	6653 (63.7)	6086 (63.7)	5467 (62.8)		1159 (64.3)	593 (65.7)	566 (62.8)	
≥30	746 (7.1)	686 (7.2)	60 (6.6)		122 (6.8)	62 (6.9)	60 (6.7)	
Income								
1st tertile (high)	2182 (20.9)	2012 (21.1)	170 (18.8)	0.0933	370 (20.5)	200 (22.2)	170 (18.9)	0.0706
2nd tertile	3337 (31.9)	3060 (32.0)	277 (30.7)		516 (28.6)	239 (26.5)	277 (30.7)	
3rd tertile	4934 (47.2)	4478 (46.9)	456 (50.5)		918 (50.9)	463 (51.3)	455 (50.4)	
Hypertension, N (%)	9200 (88.0)	8411 (88.1)	789 (87.4)	0.5731	1594 (88.4)	806 (89.4)	788 (87.4)	0.2120
Chronic kidney disease, N (%)	259 (2.5)	223 (2.3)	36 (4.0)	0.0033	50 (2.8)	14 (1.6)	36 (4.0)	0.0026
Atrial fibrillation, N (%)	119 (1.1)	97 (1.0)	22 (2.4)	0.0002	49 (2.7)	28 (3.1)	21 (2.3)	0.3848
Heart failure, N (%)	413 (4.0)	369 (3.9)	44 (4.9)	0.1621	94 (5.2)	50 (5.5)	44 (4.9)	0.5963
Duration of antiplatelet therapy (months)	50 ± 37	51 ± 37	37 ± 33	<0.0001	36 ± 32	36 ± 31	37 ± 33	0.2292
Risk associated with DM								
DM risk								
Very high-risk, N (%)	10,347 (99.0)	9457 (99.0)	890 (98.6)	0.2454	1782 (98.8)	893 (99.0)	889 (98.6)	0.5199
High-risk, N (%)	106 (1.0)	93 (1.0)	13 (1.4)		22 (1.2)	9 (1.0)	13 (1.4)	
Duration of DM (months)	60 ± 49	59 ± 48	64 ± 58	<0.0001	61 ± 54	59 ± 50	64 ± 58	<0.0001
Insulin, N (%)	90 (0.9)	81 (0.9)	9 (1.0)	0.7846	15 (0.8)	6 (0.7)	9 (1.0)	0.6041
Target organ damage, N (%)	4935 (47.2)	4524 (47.4)	411 (45.6)	0.3014	808 (44.8)	397 (44.0)	411 (45.6)	0.5382
Diabetic retinopathy, N (%)	2584 (24.7)	2372 (24.8)	212 (23.5)	0.3868	406 (22.5)	194 (21.5)	212 (23.5)	0.3379
Diabetic neuropathy, N (%)	2915 (27.9)	2654 (27.8)	261 (28.9)	0.5002	518 (28.7)	257 (28.5)	261 (28.9)	0.8759
Diabetic nephropathy, N (%)	1517 (14.5)	1397 (14.6)	120 (13.3)	0.2971	240 (13.3)	120 (13.3)	120 (13.3)	1.0000
Laboratory data								
Fasting blood glucose (mg/dL)	139 ± 38	139 ± 39	135 ± 32	0.0895	135 ± 36	136 ± 39	135 ± 32	0.1654
Total cholesterol (mg/dL)	192 ± 41	192 ± 42	189 ± 34	0.6485	190 ± 34	190 ± 33	189 ± 34	0.7266
Triglyceride (mg/dL)	173 ± 124	174 ± 126	166 ± 96	0.7143	167 ± 90	167 ± 82	166 ± 96	0.4603
LDL cholesterol (mg/dL)	109 ± 46	109 ± 47	107 ± 30	0.4151	108 ± 30	108 ± 29	107 ± 30	0.7592
HDL cholesterol (mg/dL)	51 ± 17	51 ± 18	51 ± 10	0.3596	51 ± 10	51 ± 10	51 ± 10	0.5697
Hemoglobin	14 ± 1	14 ± 1	14 ± 1	0.3156	14 ± 1	14 ± 1	14 ± 1	0.8739
Creatinine	1.0 ± 0.7	1.0 ± 0.7	1.0 ± 0.5	0.3322	1.0 ± 0.6	1.0 ± 0.7	1.0 ± 0.5	0.3972
Medication								
Statin, N (%)	8700 (83.2)	7901 (82.7)	799 (88.5)	<0.0001	1602 (88.8)	804 (89.1)	798 (88.5)	0.7089

DM: Diabetes mellitus, LDL: Low-density lipoprotein, HDL: High-density lipoprotein.

**Table 2:** Pre-matched and post-matched events by antiplatelet therapy.

	Aspirin	Clopidogrel	HR (95% CI)
NACE			
Pre-matched	1676/9550 (17.6)	171/903 (18.9)	1.78 (1.52–2.09)
Post-matched	217/902 (24.1)	170/902 (18.9)	0.97 (0.79–1.19)
Efficacy endpoint *			
Pre-matched	1511/9550 (15.8)	161/903 (17.8)	1.88 (0.60–2.22)
Post-matched	199/902 (22.1)	160/902 (17.7)	1.02 (0.82–1.26)
GI bleeding			
Pre-matched	192/9550 (2.0)	11/903 (1.2)	0.89 (0.49–1.64)
Post-matched	25/902 (2.8)	10/902 (1.1)	0.48 (0.23–1.01)
ICH			
Pre-matched	106/9550 (1.1)	15/903(1.7)	2.20 (1.28–3.79)
Post-matched	13/902 (1.4)	15/902 (1.7)	1.36 (0.64–2.88)
Myocardial infarction			
Pre-matched	46/9550 (0.5)	2/903 (0.2)	0.65 (0.16–2.69)
Post-matched	5/902 (0.6)	2/902 (0.2)	0.52 (0.10–2.83)
Stroke			
Pre-matched	100/9550 (1.1)	17/903 (1.9)	2.62 (1.57–4.40)
Post-matched	16/902 (1.8)	16/902 (1.8)	1.22 (0.60–2.48)
All-cause death			
Pre-matched	1445/9550 (15.1)	158/903 (17.5)	1.87 (1.58–2.20)
Post-matched	190/902 (21.1)	157/902 (17.4)	1.07 (0.86–1.33)

Matched: Propensity score-matched, NACE: Net adverse clinical event, GI: Gastrointestinal bleeding, ICH: Intracranial hemorrhage, HR: Hazard ratio, CI: Confidence interval. * Efficacy endpoint: composite of myocardial infarction, incident stroke, and all-cause death.

## Data Availability

The datasets analyzed in the current study can be requested from the NHIS upon reasonable justification (https://nhiss.nhis.or.kr/en/z/a/001/lpza001m01en.do, accessed from 10 October 2024, to 15 April 2025).

## Abbreviations

The following abbreviations are used in this manuscript:

ASCVD	Atherosclerotic cardiovascular disease
BARC	Bleeding Academic Research Consortium
BMI	Body mass index
CAPSTONE-DM	Clopidogrel versus AsPirin as a STrategy fOr the PreventioN of cardiovascular diseasEs in patients with Diabetes Mellitus
GI	Gastrointestinal
ICH	Intracranial hemorrhage
MI	Myocardial infarction
NACE	Net adverse clinical event
NHIS	National Health Insurance System
NHIS-NSC	National Health Insurance System National Sample Cohort
PCI	Percutaneous coronary intervention
PSM	Propensity score matching
